# FcεRI: A Master Regulator of Mast Cell Functions

**DOI:** 10.3390/cells11040622

**Published:** 2022-02-11

**Authors:** Yuka Nagata, Ryo Suzuki

**Affiliations:** Faculty of Pharmaceutical Science, Institute of Medical, Pharmaceutical and Health Sciences, Kanazawa University, Kakuma-machi, Kanazawa 920-1192, Japan; nagata@p.kanazawa-u.ac.jp

**Keywords:** allergy, antigen, IgE, FcεRI, mast cell, desensitization

## Abstract

Mast cells (MCs) perform multiple functions thought to underlie different manifestations of allergies. Various aspects of antigens (Ags) and their interactions with immunoglobulin E (IgE) cause diverse responses in MCs. FcεRI, a high-affinity IgE receptor, deciphers the Ag–IgE interaction and drives allergic responses. FcεRI clustering is essential for signal transduction and, therefore, determines the quality of MC responses. Ag properties precisely regulate FcεRI dynamics, which consequently initiates differential outcomes by switching the intracellular-signaling pathway, suggesting that Ag properties can control MC responses, both qualitatively and quantitatively. Thus, the therapeutic benefits of FcεRI-targeting strategies have long been examined. Disrupting IgE–FcεRI interactions is a potential therapeutic strategy because the binding affinity between IgE and FcεRI is extremely high. Specifically, FcεRI desensitization, due to internalization, is also a potential therapeutic target that is involved in the mechanisms of allergen-specific immunotherapy. Several recent findings have suggested that silent internalization is strongly associated with FcεRI dynamics. A comprehensive understanding of the role of FcεRI may lead to the development of novel therapies for allergies. Here, we review the qualitatively diverse responses of MCs that impact the attenuation/development of allergies with a focus on the role of FcεRI toward Ag exposure.

## 1. Introduction

Recently, allergies have emerged as a public health problem, as over one-quarter of the population in industrialized countries is affected and the prevalence of allergies is increasing [1]. Pathophysiological features of allergy are characterized by a diverse set of clinical symptoms, and the disease burden reflects the long-term consequences of chronic allergic inflammation at sites of persistent or repetitive exposure to antigens (Ags) [2]. Different phenotypes/endotypes have been recognized with the recent increase in the heterogeneity of Ag contents [3].

Mast cells (MCs) initially respond rapidly to exogenous Ags, which is the principal initial effector immune response involved in modulating allergic inflammation [2,4,5]. Ag-induced MC degranulation is the basis of anaphylaxis and other severe allergic reactions [6]. Ag binding to immunoglobulin E (IgE) that is bound to its receptor (FcεRI) causes the crosslinking of IgE–FcεRI complexes, resulting in the release of biologically active mediators such as histamine, serotonin, and leukotrienes within minutes. Cytokines, chemokines, and growth factors that are transcriptionally upregulated in MCs are secreted over a period of hours after initial MC activation, leading to prolonged inflammation [7,8].

FcεRI, the high-affinity IgE receptor, distinguishes Ag–IgE interactions and drives cellular allergic responses [9]. FcεRI is a unique molecular target that initiates different functional outcomes of MC responses and allergic inflammation [10,11]. In this review, we highlight recent advances in the understanding of FcεRI as a master regulator of MC functions, with a focus on FcεRI behavior in response to various Ags and subsequent cellular responses, including desensitization, which could be a potential strategy for alleviating allergies.

## 2. FcεRI

### 2.1. Crosslinking of the IgE–FcεRI Complex with Antigen

The high-affinity IgE receptor, FcεRI, is primarily expressed on MCs, basophils, and Ag-presenting cells, and mainly exists as the heterotetramer αβγ2. However, there are differences among species; an alternate trimeric form αγ2 is expressed on human, but not rodent. The αβγ2 consists of a single-membrane-spanning α-subunit that contains the IgE-binding domain, a tetra-spanning β-subunit that contains a single immunoreceptor tyrosine-based activation motif (ITAM), and two γ-subunits that exist as a disulfide-linked homodimer and also contain one ITAM each. The importance of the α-subunit in the FcεRI-mediated allergic reaction was demonstrated by the absence of allergic reactions in α-subunit-deficient mice [12]. The FcεRIα binds to the Fc fragment of IgE at a 1:1 ratio to form the IgE–Fc complex. Two hydrophobic regions in Cε3 of the IgE–Fc complex asymmetrically bind to two Ig-like extracellular domains of the α-subunit. This asymmetric interaction follows a 1:1 stoichiometry, and both distinct integrations increase the binding avidity, leading to high-affinity binding [13,14,15].

Multivalent Ags cause cross-linking of the IgE–FcεRI complex, triggering signaling cascades that result in the release of preformed mediators stored in granules. In response to Ag, Lyn kinase phosphorylates the ITAMs of FcεRIβ and FcεRIγ, which initiates a complex signaling cascade involving a series of membrane-associated and cytoplasmic signaling molecules [8,16,17,18]. Furthermore, bindings of monomeric IgE to FcεRI, in the absence of Ags, is capable of inducing phosphorylation of signaling molecules and MC responses (MC survival and granules maturation) without degranulation [19].

### 2.2. FcεRI Clustering: Size, Stability, and Mobility Dynamics

In the cell membrane, the IgE–FcεRI complex diffuses freely under resting conditions. Multivalent Ag binding to IgE leads to the reorganization of FcεRI into large aggregates on the cell surface within seconds to minutes, which causes a transition in the receptor from a diffuse state to a highly immobile state [20]. This behavior of the FcεRI cluster, including its mobility, kinetics, and size, has been highlighted as a potential feature that governs signal initiation. The relationship between the FcεRI cluster size and mobility has been clearly demonstrated in rat basophilic leukemia (RBL-2H3) cells and bone marrow-derived MCs (BMMCs). The imaging studies using quantum dot (QD)-based probes revealed that the small aggregates remain mobile on cell surface, whereas there is abrupt immobilization in large aggregates, where the actin cytoskeleton plays an important role in cross-link-induced immobilization of FcεRI [21,22]. Andrews et al. showed that Ag-induced FcεRI immobilization was required for receptor internalization, but not for signal transduction, and they presented evidence that FcεRI immobilization could cause signal termination. Although the small FcεRI aggregates induced by sub- and supra-optimal concentrations/Ag valency did not lead to strong degranulation, FcεRIβ phosphorylation was detectable, indicating that signaling was competent. They concluded that the size of receptor clusters regulates mobility, signaling competence, and FcεRI internalization [22].

The same group also investigated the spatial/density regulation of the FcεRI clusters of MC, and they demonstrated that the FcεRI redistribution in regions of the membrane after Ag stimulation is also remarkable for the complexity of the FcεRI dynamics and signaling initiation. Sequential images of IgE–FcεRI complexes, assessed by total internal reflection fluorescence (TIRF) microscopy, have been used to observe the dynamics of receptor redistribution upon contact with the ligand. Importantly, Carroll-Portillo et al. demonstrated that small mobile clusters coalesced into large, cholesterol-rich patches that occupied the central portion of the contacting membrane and are likely composed of smaller aggregates in the same region, suggesting that dense populations of mobile receptors could initiate low-level degranulation [23]. These findings highlight the importance of FcεRI dynamics not only in the temporal behavior of internalization but also in membrane topography. Furthermore, they investigated the topographical details of IgE–FcεRI complexes on the plasma membrane at the nanoscale level using high-resolution microscopy [24]. They observed a coalesced large patch, termed a signaling patch, and confirmed signaling competency downstream of the patch [24]. These findings contribute to the understanding of the sophisticated signaling mechanisms associated with FcεRI and seem to be indicative of relationships between receptor diffusion, clustering, signal initiation, and MC activation. Additionally, a quantitative study was performed to measure FcεRI motion within clusters and coalesced patches using single-particle tracking, the results of which provided significantly support for a mechanism whereby FcεRI clusters diffuse and then coalesce to form large patches in specific regions of the plasma membrane [25].

These examples illustrate the importance of FcεRI dynamics and subsequent cellular responses at a low Ag concentration or valency, which in turn drive the formation of small and mobile FcεRI clusters that were previously considered a simple and diminished (i.e., weak) response. Signaling diversity is significantly related to FcεRI dynamics. Src-homology-2-containing inositol phosphatase (SHIP) is a negative regulator of MCs [26], and its relationship with small FcεRI cluster formation has been proposed [27]. Mahajan et al. performed experiments with a new structurally defined trivalent ligand, DF3, and clearly showed the significant participation of SHIP1 in the small FcεRI cluster, which forms under low-concentration and low-valency conditions [27]. Furthermore, the signaling consequences of differential FcεRI aggregation were investigated through continuous stimulation of RBL-2H3 cells with DF3. The results revealed that Syk and SHIP1 drove rapid short-lived positive signals and slower long-lived negative signals, respectively [28], and time-dependent signaling following stimulations that likely operate during differential FcεRI cluster initiation [28]. These timescale observations further strengthen the view that FcεRI dynamics may regulate signaling events and initiate cellular outcomes. Recent findings from Bag et al. demonstrated a direct relationship between the mobility of small or large clusters and the formation of lipid raft domains [29]. Importantly, they found that stabilized liquid ordered (Lo)-like nanodomains around the FcεRI cluster (which are accessible by Lyn kinase, but not transmembrane phosphatase), provide essential spatial filtration that augments Lyn binding and FcεRI phosphorylation, while suppressing dephosphorylation by phosphatases [29].

Those studies were performed using concentration- and valency-adjusted agents. The Ag properties and their interactions with IgE are important for determining FcεRI dynamics and the initiation of subsequent cellular reactions, as discussed below in Section 2.3.

### 2.3. Antigen Properties: Concentration, Valency, and Affinity

Naturally encountered allergens are typically complex and structurally heterogeneous proteins with multiple epitopes [30]. Moreover, naturally occurring IgEs display the heterogeneity of antibodies with different affinities against Ags [31]. In a recent study, naturally occurring pollen allergens (not synthetic allergens) were studied to demonstrate the complexity of allergen sources and investigate their interaction with IgE [32]. Earlier studies identified a variety of olive pollen allergens [33] and house dust mite allergens [34,35], whose variations are recognized as isoallergens. These characteristics contribute to the overall complexity of allergens and the variety of associated disease symptoms. The magnitude of the MC responses indeed depends on the allergen properties. The concentrations, valencies, and affinities of Ags affect the interaction with IgE and MC responses, which may cause complex allergic responses.

The influence of the Ag concentration on FcεRI dynamics has been elucidated; degranulation in MCs displays a remarkable bell-shaped response with increasing Ag concentrations [36,37]. This phenomenon is a particular feature of FcεRI and differs from many other receptors that reach a plateau phase in response to high ligand concentrations. The suppressed responses at supra-optimal concentrations have been suggested to be not due to reduced FcεRI crosslinking [38,39]. There is a FcεRI-mediated active turn-off mechanism in the descending portion of the specific bell-shaped degranulation responses in MCs (reviewed in [40]). Indeed, MC responses to supra-optimal Ag concentrations are not simply weaker than those at the optimal Ag concentration. Ca^2+^ mobilization in MCs, which results from intracellular Ca^2+^ release from the endoplasmic reticulum (ER) and subsequent extracellular Ca^2+^ influx, is important for degranulation. Comparing the intracellular Ca^2+^ responses under optimal and supra-optimal concentration stimulation demonstrated markedly different Ca^2+^-mobilization patterns [36]. In particular, Ca^2+^ release from the ER was slightly stronger at a supra-optimal Ag concentration. The supra-optimal Ag concentration resulted in a transient increase in intracellular Ca^2+^, whereas the optimal Ag concentration showed a sustained pattern [36], suggesting that active negative regulation occurs. Consistently, the results of that study also revealed that protein tyrosine phosphorylation was stimulated slightly more with a supra-optimal Ag concentration the optimal Ag concentration. This negative signaling was mainly mediated by the activation of SHIP by Lyn [36]. However, the precise mechanisms of SHIP recruitment remain under investigation. One accepted mechanism is that FcεRIβ can interact with SHIP1 via its unique ITAM sequence upon supra-optimal stimulation [17,41]. The results of another recent study demonstrated that stimulating IgE-sensitized MCs with a supra-optimal Ag concentration induced FcγRIIb tyrosine phosphorylation in an IgG-independent manner [42]. Intriguingly, the co-aggregation of FcεRI and FcγRIIb was associated with a submembranous F-actin meshwork that negatively interfered with MC activation at supra-optimal Ag concentrations [42]. Taken together, these data suggest that tightly controlled cellular reactions may reflect a complementary mechanism that activates or compensates for allergic responses to different intensities of stimulation.

The Ag valency is also an important factor in controlling the signal intensity as well as Ag concentration. Naturally occurring allergens are the most common complex type of multivalent proteins. Despite the small size of the timothy grass pollen allergen, Phl p 5, it is known to be highly allergenic and has multiple independent IgE epitopes [32,43]. In experimental settings, multivalent agents such as DNP-BSA are commonly used to crosslink IgE–FcεRI to enhance MC activation as a potent stimulation. The valency and concentration-dependent characteristics (size and mobility) of FcεRI clusters have been demonstrated, as discussed above [22,24]. Typically, high-valence Ags rapidly form large and immobile FcεRI clusters. Monovalent Ags generally cannot form aggregates with FcεRI, but can stimulate FcεRI mobilization/redistribution and form a distinct kind of FcεRI patch, termed a signaling patch [23]. Monovalent Ags are capable of competitive inhibition that rapidly influences the binding of multivalent ligands to receptors; thus, monovalent Ags are considered candidates for therapeutic interventions targeting FcεRI. A previous study involving transmission electron microscopy and real-time TIRF imaging of FcεRI in monovalent Ag-stimulated RBL-2H3 cells primed with fluorescent-labeled IgE showed significantly different FcεRI membrane topographies between multivalent and monovalent ligands [23]. In that study, the authors determined the Ag mobility by engaging anti-DNP IgE on the surfaces of cells presenting either a monovalent DNP-embedded lipid bilayer (mobile ligand) or a chemically crosslinked multivalent DNP (immobilized ligand). Immobile multivalent DNP_24_-BSA stimulation resulted in the rapid formation of stable, moderately sized clusters with typical levels of RBL-2H3 degranulation. The mobile monovalent ligand showed a high density of mobile receptors, in which thousands of mobile receptors were densely packed in the centralized region of the ventral membrane, despite the lack of direct crosslinking [23]. With regard to the un-crosslinked IgE–FcεRI bearing, several studies using IgE clones indicated/discussed mechanisms underlying the effect of IgE in the absence of Ags [44,45], which have been proposed that the IgE–FcεRI could be clustering and internalize without Ag, suggested that the importance of Fab regions of IgE for triggering the IgE-mediated robust activation of MCs (reviewed in [46]). Although the mechanism of un-crosslinked internalization of FcεRI is still being investigated, such studies will likely be found to have important implications for further characterization of the size, mobility, and density of FcεRI clusters.

**Figure 1:**
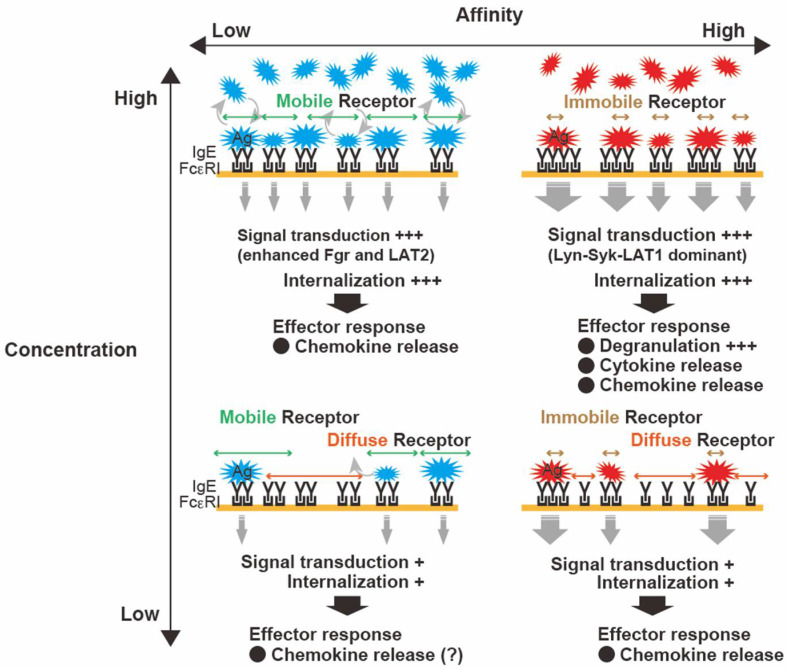
Characteristics of antigen (Ag)-dependent control of clustering of the high-affinity IgE receptor (FcεRI) and subsequent mast cell (MC) responses. The characteristics of Ags (i.e., concentration and affinity) regulate FcεRI functions. High-affinity Ags elicit robust bridging between immunoglobulin E (IgE) and large immobile clusters with increasing Ag concentration, resulting in typical MC activation. In contrast, low-affinity Ags likely allow FcεRI to remain mobile and diffuse, leading to slower internalization (even at high Ag concentration) within FcεRI signalosomes. These differential dynamics of FcεRI may represent a characteristic feature that translates Ag properties into differential outcomes.

FcεRI functionally distinguishes differences in the affinity of IgE antibodies for different Ags. Naturally occurring polyclonal IgEs show heterogeneity with different affinities against Ags [47]. Differences in binding affinities are also known as differences in binding kinetics. Low-affinity Ags dissociate IgE from FcεRI more rapidly than high-affinity Ags. A comparison of MC responses to a high-affinity Ag (dinitrophenyl (DNP)-Ag) and a low-affinity Ag (nitrophenyl (2NP)-Ag) demonstrated that the quantitative difference in the affinities between the IgE and Ag determines the quality of subsequent cellular reactions [48]. The effects of DNP-Ag and 2NP-Ag were compared at concentration that elicited similar FcεRI phosphorylation levels. Although both Ags elicited similar degrees of FcεRI phosphorylation, the behavior of FcεRI clusters (size, mobility, and distribution) and MC secretory responses (cytokines and chemokines) differed significantly. Live-imaging analysis of FcεRI dynamics revealed that 2NP-Ag induced slowed mobility of FcεRI clusters, whereas DNP-Ag treatment resulted in rapidly formed FcεRI clusters. These differences in FcεRI dynamics were found to be related to distal signaling and functional responses. DNP-Ag elicited a more robust linker of activated T cells 1 (Lyn-Syk-LAT1)-dependent signaling, which led to the release of inflammatory cytokines (e.g., TNF-α, IL-6, and IL-13). In contrast, 2NP-Ag dominantly released chemokines (e.g., CCL2, CCL3, and CCL4) through a pathway that depended on the Src-family kinase Fgr and linker for activation of T cell family member 2 (Fgr-LAT2). A mouse model of passive cutaneous anaphylaxis showed a greater magnitude of skin inflammation with DNP-Ag than with 2NP-Ag. DNP-Ag-stimulated MCs recruited neutrophils to the site of inflammation, whereas 2NP-Ag-stimulated MCs recruited monocytes/macrophages (reviewed in [49]). The role of differential recruitment of neutrophils or monocytes/macrophages remains under investigation, the clarification of which may represent a considerable advance in understanding the regulation of systemic allergic inflammation. The mechanisms responsible for different responses to DNP-Ag or 2NP-Ag, together with other evidence pertaining to FcɛRI dynamics, suggest that low-affinity Ags-stimulated FcɛRI clusters might remain mobile/diffuse and can induce MC responses (Figure 1).

This information may help in developing novel strategies for controlling FcεRI clustering in MCs and the subsequent allergic responses. A recent report by Mahajan et al. demonstrated that not only the valency and concentration of an allergen, but also the FcεRI occupancy with IgE, help set the thresholds for MC effector responses [50]. High-affinity IgEs for multimeric Ags effectively induce degranulation. In contrast, low-affinity IgEs can mediate effector responses via the avidity effect of polyvalent Ags [32]. A complicated input is likely governed by Ags and IgEs, whereas FcεRI may dynamically respond to the stimulation intensity and translate it into an appropriate response. Many researchers have investigated signaling events and MC secretory responses in response to high affinity antigen with different concentrations. Although there is still being uncovered roles of low affinity Ags on MC responses, differential behavior of FcɛRI under various concentration of low affinity Ags appears to alter MC responses and disease outcome.

### 2.4. Perspective

FcεRI displays diverse dynamics (i.e., receptor-cluster size, mobility, distribution, and naturally occurring allergens) and IgEs show heterogeneous properties. The functional responses of FcεRI may be studied to decipher the characteristics of endogenous Ags and/or their interactions with IgEs, and to regulate MC-dependent allergic responses, thereby enabling further elucidation of the clinical relevance of human allergic responses.

## 3. FcεRI: Therapeutic Potential and Benefits

### 3.1. Inhibition of IgE–FcεRI Binding

FcεRI has been targeted for clinical benefits to inhibit or attenuate MC degranulation, and effective clinical approaches for suppressing IgE-dependent MC activation have emerged [51,52]. Omalizumab, a humanized anti-IgE monoclonal antibody that binds to the Cε3 domain of IgE, can prevent the binding of circulating IgE to FcεRI, is a common and preferred treatment (especially for chronic urticaria), and is effective against asthma [53,54,55]. Omalizumab binds free IgE, disrupts IgE–FcεRI complexes, and decreases FcεRI expression [56,57]. IgE binding to FcεRI further increases expression of the receptor, resulting in stable FcεRI expression on the MC surface [58,59]. Omalizumab does not crosslink FcɛRI-bound IgE on the MC surface because of conformational changes in FcεRI-bound IgE that mask omalizumab-binding sites [54,60]. Omalizumab could dissociate/remove IgE from FcɛRI; however, only poor disruptive activity has been observed [61,62]. Although the precise mechanisms involved in stripping IgE from FcɛRI remain to be elucidated, allosteric destabilization and facilitated dissociation of the IgE–FcɛRI complex have been suspected as potential mechanisms [63,64]. Additionally, omalizumab has been studied as an adjuvant therapy for oral immunotherapy (OIT) against food allergies [65]. Open-label pilot studies in cow’s milk and peanuts allergy suggested that 9–12 weeks of omalizumab therapy could facilitate rapid oral desensitization to each allergen in a high-risk patient [66,67]. The mechanisms of the combined therapy of OIT plus omalizumab have been studied. Recent study suggested that the combined therapy promotes allergen desensitization through an initial omalizumab-dependent acute depletion of allergen-reactive effector T cells, and which is followed by an increase in allergen-specific regulatory T (Treg) cell activity due to the reversal of their T helper 2 (Th2) cell-like program [68]. Regarding other antibody antagonism/antibody neutralization, ligelizumab is another potent humanized anti-IgE monoclonal antibody that binds to free IgE with a higher affinity than omalizumab. Ligelizumab binds to the C3 domain of IgE, but unlike omalizumab, it can also bind to IgE-bound to CD23, a low-affinity IgE receptor expressed on B cells [51,69]. In 2020, Gasser et al. demonstrated the structural and mechanistic differences between ligelizumab and omalizumab, where they report epitope differences between the anti-IgE antibodies and indicate the differences contribute to their qualitatively distinct IgE-receptor inhibition profiles [69].

DARPins are small molecules and highly stable non-antibody protein scaffolds. Anti-IgE DARPins, which are genetically engineered proteins comprising a varying number of stacked ankyrin-repeat domains, also block IgE binding to FcεRI. Ankyrin-repeat domain binding can affect the stability and effector functions of target proteins. The motivation for engineering DARPins was to generate binding proteins that can be used to target proteins with high affinity and specificity, essentially replacing the use of monoclonal antibodies [51,70]. Therefore, the advantage of anti-IgE DARPins is that they not only neutralize free IgE but also actively disrupt pre-formed IgE–FcεRI complexes through a facilitated dissociation mechanism [63,71]. The disruption of IgE–FcɛRI complexes by anti-IgE DARPins has been shown to be 10,000-fold more efficient than omalizumab in both in vitro and ex vivo human tissues [61]. Recently, newly engineered highly potent disruptive IgE inhibitors have been developed based on DARPin scaffolds, and their efficacies has been assessed [72].

Natural products, including herbal medicines and nutritional supplements, have been reported to inhibit MC degranulation. The underlying mechanisms appear to involve the blockade of IgE–FcεRI binding or IgE-mediated FcεRI signaling. For example, carotenoids suppress Ag-induced FcεRI aggregation and inhibit FcεRI-mediated intracellular signaling [73]. Procyanidin-enriched extracts from apples inhibit IgE binding to FcεRI and its subsequent signaling [74,75]. Medicinal herbs, such as *Rubia cordifolia* and *Dianthus superbus* in China; KOTMIN13 (composed of *Inula japonica* flowers), *Trichosanthes kirilowii* semen, *Peucedanum praeruptorum* radix, and *Allium macrostemon* bulbs in Korea [76]; and *Paeonia* radix and *Zanthoxyli* fructus in Japan, suppress MC degranulation and murine allergic inflammation [77,78]. These observations highlight the potential of plants and herbs as sources of bioactive compounds against allergic responses. However, their precise mechanisms are not fully understood, especially in the context of regulating FcεRI dynamics. We previously reported the potential of a natural product for regulating FcεRI functions; an ephedra herb extract, Mao, in Japanese, significantly suppressed Ag-induced MC degranulation [79]. Mao significantly induced FcεRI internalization in MCs, thereby inhibiting Ag/IgE-dependent MC degranulation. We also showed that Mao induced FcεRI reorganization into distinct small clusters, without degranulation. These effects in Mao-treated MCs mimicked Ag-induced rapid desensitization states, suggesting that Mao has both therapeutic potential and unknown mechanisms, specifically in terms of FcεRI cluster formation, which might be involved in MC desensitization [79].

### 3.2. FcεRI–FcγRIIb Coaggregation and Inhibitory Signal against FcεRI

Inhibitory signaling against FcεRI is another candidate for FcεRI-targeting therapy, which is mediated by co-crosslinking ITIM-containing inhibitory IgG receptor (FcγRIIb) with FcεRI. FcγRIIb is highly expressed in murine MCs, murine basophils, and human basophils. Previous findings demonstrated that MCs isolated from human skin only express the activating FcγR and FcγRIIa proteins, whereas human cord blood-derived cultured MCs only express FcγRIIb [80,81], implying potential heterogeneity among human MCs with respect to the expression of FcγRs [82]. FcεRI and FcγRIIb were co-crosslinked separately in Ag-independent and Ag-dependent manners. Ag-independent crosslinking of these receptors was achieved with bispecific antibodies against FcεRI and FcγRIIb or Fcε–Fcγ fusion proteins to prevent allergic reactivity [83,84]. In contrast, specific IgG antibodies can induce allergen-specific IgE–FcεRI–FcγRIIb crosslinking in the presence of specific allergens and competitively inhibit allergen binding to the specific IgE [85]. A recent clinical trial of cat allergen-specific IgG cocktails for respiratory allergies showed the efficacy of therapeutic applications of IgG, and demonstrated that passive immunization with allergen-specific IgG monoclonal antibodies potently suppresses allergic symptoms. Although receptor-mediated inhibition has not been clarified in the literature, these inhibitory effects might be due to IgG-FcγRIIb-dependent and/or IgG works as a neutralizing antibody for allergen [86].

### 3.3. FcεRI Internalization in Allergen-Induced FcεRI Desensitization

Allergen-induced FcεRI desensitization, which is achieved by sequentially increasing allergen stimulation from a suboptimal concentration and is accompanied by FcεRI internalization without MC (basophil) activation, is an important mechanism underlying allergen-specific immunotherapy [87]. This is the only disease-modifying therapy/intervention available for the treatment of allergies. Successful allergen-specific FcεRI desensitization renders these effector cells less responsive or non-responsive, and the altered magnitude of mediators released from MCs represents the first step in a series of events following the induction of therapy. This could eventually lead to immunological tolerance to a specific allergen by modulating the subsequent development of Th2-biased responses. Such therapy provides a measurable benefit and is currently the only potential treatment for allergies. However, depending on disease phenotypes, the safety and efficacy are not sufficient in terms of the risk of undesirable adverse effects and the long duration of therapy [88]. Data from a recent clinical study showed a low compliance rate by patients over long periods of allergen-specific immunotherapy [89]. The authors of that study demonstrated that the compliance rates in the third year of treatment were 58.7% among patients diagnosed with allergic rhinitis (with or without allergic asthma) who were on subcutaneous immunotherapy, and 11.6% among those on sublingual immunotherapy [89]. These obvious limitations of allergen-specific immunotherapy have driven research toward alternative strategies. Several efforts aimed at improving therapeutic efficacies have been developed, including novel routes of immunotherapy, combining Ags with immunostimulatory adjuvants, and the use of modified Ags. For example, several combined therapies, such as omalizumab plus OIT, have been studied for treating food allergies. Traditional herbal medicines, which potently induce intestinal Treg cells, have been proposed as potential agents for improving the therapeutic efficacy when combined with specific Ags [90,91,92,93]. In experimental models of food allergy, combined therapies using herbal medicine suppressed MC activation at the inflammatory site and increased the therapeutic efficacy compared to OIT alone [92,93].

**Figure 2:**
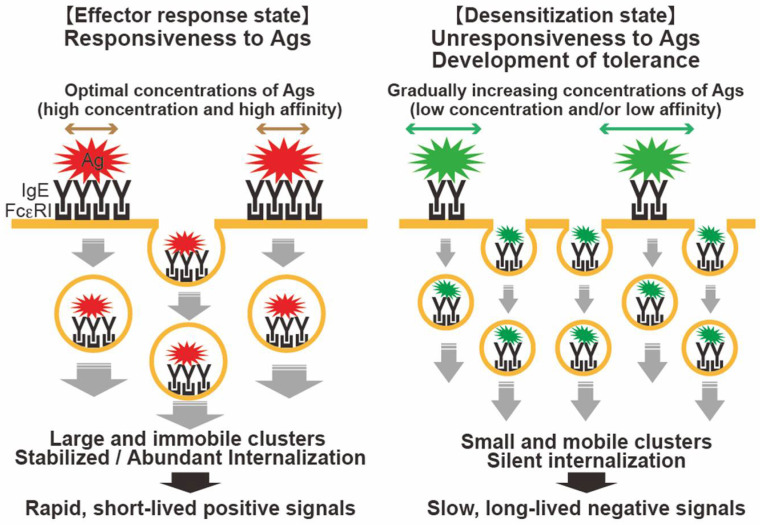
FcεRI desensitization and hypothesized mechanism of silent FcεRI internalization. Ag–IgE–FcεRI complexes are capable of internalization without MC activation. (**Left**) High-intensity stimulation (e.g., modest to high concentrations of high-affinity Ag) causes a rapid transition of FcεRI from a diffuse state to a highly immobile state, resulting in the formation of large aggregates. The rapidly internalized FcεRI clusters are observed obviously large, in which rapid short-lived positive signals are derived. (**Right**) During FcεRI desensitization with incremental Ag stimulation, suboptimal stimulations might allow FcεRI to remain mobile and diffuse, and form smaller clusters, followed by slower internalization. There is no considerable degranulation, but a sufficient degree of internalization and signals may result.

Modifying the properties of Ags has also been recognized as a potential strategy for improving immunotherapeutic efficacies [94]. A wide variety of structurally modified Ags have been generated, including chemically modified [95], adjuvant-bound [96], and recombinant hypoallergenic Ags (based on modifying the sequence of wild-type Ags) [97]. A recent study of the shrimp allergen tropomyosin showed that modifying tropomyosin by glycation decreases its allergenicity, where hypoallergenic glycated tropomyosin suppressed MC responses and allergic symptoms [98]. These modified Ags were designed to reduce adverse effects while maintaining immunogenicity at the systemic level. With regard to the local cellular events in MCs, these ideal Ags do not provoke robust MC activation, but induce FcεRI internalization. This silent FcεRI internalization could be attributed to a certain degree of FcεRI dynamics in the context of the above-mentioned findings. Desensitized MCs, whose FcεRI dynamics likely represent small and mobile FcεRI clusters and slow internalization (Figure 2), are discussed further below. Although limited data are available regarding the relationship between the desensitization efficacy and FcεRI dynamics, some novel mechanisms responsible for controlling FcɛRI dynamics based on the above studies may provide important information for improving the efficacy of FcɛRI desensitization.

To explore the mechanisms underlying FcεRI desensitization in MCs and cell-specific local responses during allergen-specific immunotherapy, in vitro desensitization studies have been performed by several different groups [99,100,101]. IgE-sensitized MCs were subjected to sequential stimulation with incrementally increasing concentrations of Ag, which started from a subthreshold concentration and reached an optimal level. Desensitized MCs show decreases in IgE-bound FcεRI, which could be explained by internalization of the IgE–FcεRI complex [99,100]. Ang et al. investigated aberrant rearrangements of cytoskeletal actin fibers that inhibited FcɛRI-mediated Ca^2+^ flux and intracellular vesicle tracking [101]. Furthermore, recent data demonstrated activated tolerogenic capacities in the desensitized state [102] and that desensitized MCs showed tolerogenic features by releasing anti-inflammatory cytokines during the desensitization process, which were suggested to induce Treg cells in the local inflammatory site [102]. However, the precise mechanisms of successful desensitization, especially in the context of desensitized FcεRI dynamics, have not been thoroughly investigated. We recently performed in vitro MC desensitization using both DNP-HSA and ovalbumin (OVA) Ags and investigated the formation and localization of FcεRI clusters in MCs. We demonstrated that FcεRI clusters in in vitro desensitized MCs were significantly smaller, despite sufficient FcεRI internalization as a consequence of desensitization [79,103]. The small FcεRI clusters in desensitized MCs may be mobile and slowly internalized. Additionally, when we determined the Ag properties (i.e., antigenicity) using heated OVA and naïve OVA, heated OVA showed higher allergenicity, possibly because the thermal treatment caused OVA aggregation. Heated OVA failed to induce desensitization at the same concentration as naïve OVA. Heated OVA showed undesirable degranulation during desensitization, where significantly larger clusters were formed [103]. Collectively, these results suggest that manipulation of the allergenicity is critical for successful desensitization.

Taken together, these data reviewed above indicate that specific features of FcεRI dynamics appear to be involved in FcεRI desensitization in MCs. With therapeutic interventions based on FcεRI desensitization, ideal Ags may be those that can flexibly and generally induce small FcεRI clusters and regulate moderate FcεRI dynamics, which may contribute to the appropriate regulation of FcεRI clusters that are small and may be mobile, diffuse, and compete for internalization (Figure 2). However, one important limitation/question is whether small cluster induction and internalization, which certainly prevent rapid and robust degranulation, might induce other types of signaling or sustained/prolonged immunological responses. Indeed, the MC response shows diverse reactivity upon weak stimulation [104]. Investigating such mechanisms may lead to discoveries that influence effective desensitization and produce more reliable information. Further development of this topic might help in understanding the regulatory mechanisms involved in related therapies, which may offer insights into optimizing Ag immunotherapy and rapid desensitization strategies.

### 3.4. Perspective

Adequate regulation of FcεRI dynamics is a potential intervention strategy for controlling MC qualitative functions and allergic inflammation. FcεRI may actively adjust cellular responses even when no considerable degranulation occurs immediately after Ag challenge, which is likely to contribute to improving FcεRI targeting therapy.

## 4. Conclusions

FcεRI displays dynamic changes in response to the properties of Ags and IgEs, which induce differential signaling pathways that regulate MC functions. A complementary mechanism may exist, even after weak or simple input patterns. FcεRI on the MC responds to the stimulation intensity/quality and translates it into an appropriate MC response. Taking advantage of this FcεRI characteristic may improve FcεRI-targeting therapeutics, especially FcεRI desensitization and Ag-specific immunotherapy for allergies.

## Data Availability

The data presented in this review are available on request from the corresponding author.
